# Safety and Oncologic Outcomes of Robotic Lobectomy in the Early Adoption Phase: First Single-Surgeon Experience from the Polish Healthcare System

**DOI:** 10.3390/cancers18071115

**Published:** 2026-03-30

**Authors:** Wojciech Migal, Michał Wiłkojć, Agnieszka Majewska, Maciej Walędziak, Krzysztof Karol Czauderna, Anna Różańska-Walędziak

**Affiliations:** 1 Faculty of Medicine, Collegium Medicum, Cardinal Stefan Wyszynski University in Warsaw, 01-815 Warszawa, Poland; w.migal@student.uksw.edu.pl (W.M.); a.majewska@student.uksw.edu.pl (A.M.); 2 Department of General, Oncological, Metabolic and Thoracic Surgery, Military Institute of Medicine—National Research Institute, 04-141 Warszawa, Poland; 3 Medical Faculty, University of Gdańsk, 80-210 Gdańsk, Poland; krzysztof.czauderna@gumed.edu.pl; 4 Department of Human Physiology and Pathophysiology, Faculty of Medicine, Collegium Medicum, Cardinal Stefan Wyszynski University in Warsaw, 01-815 Warszawa, Poland; aniaroza@tlen.pl

**Keywords:** robotic-assisted thoracic surgery, robotic lobectomy, non-small cell lung cancer, perioperative outcomes, Polish healthcare system, early program implementation

## Abstract

Background: Robotic-assisted thoracic surgery is increasingly recognized as an advanced minimally invasive technique for treating non-small cell lung cancer, offering technical advantages such as enhanced precision and visualization. Although numerous studies have been published worldwide, there are no comparable data from Poland. Therefore, evidence on the perioperative safety and oncologic adequacy of robotic-assisted lobectomy during early phase of program implementation within the Polish healthcare system remains limited. Methods: This retrospective, single-institution observational study included 81 consecutive patients who underwent robotic-assisted lobectomy for primary NSCLC between January 2022 and December 2024. All procedures were carried out using the da Vinci Xi system with a standardized four-arm portal approach. Clinical, perioperative, and pathologic parameters were prospectively collected and analyzed descriptively. Postoperative complications were classified according to Clavien-Dindo. Results: The median patient age was 70 years (IQR: 65–74), 52% were male, and 67% had a history of smoking. Adenocarcinoma was the predominant histologic subtype (51%). The median operative time was 176 min (IQR: 149–220). There were no conversions to thoracotomy and no 30-day mortalities. Postoperative complications occurred in 24% of cases, with prolonged air leak being most common (17%). The median hospital stay was 8 days (IQR: 6–10). R0 resection was achieved in 96% of patients, with a median of 14 lymph nodes dissected across 5 nodal stations. Conclusions: Robotic-assisted lobectomy performed during the early implementation phase of a national program demonstrated low morbidity, high rates of complete (R0) resection, and adequate lymph node yields consistent with international benchmarks. These results support the feasibility of robotic lobectomy within the Polish healthcare setting; however, the single-surgeon, single-center design limits generalizability. Further multicenter prospective studies are needed to confirm reproducibility, assess learning curves, and evaluate long-term oncologic outcomes.

## Simple Summary

Robotic-assisted surgery is a modern, minimally invasive technique that may improve the precision and safety of lung cancer operations. Although this approach is increasingly used worldwide, there is still limited information about its use in Poland, especially during the early stages of introducing such technology into routine clinical practice. The aim of this study is to evaluate the safety and effectiveness of robotic-assisted lung lobectomy in patients with non-small cell lung cancer treated at a Polish center during the initial phase of a robotic surgery program. By analyzing surgical outcomes, complications, and cancer-related results, the authors seek to determine whether this technique can be safely implemented within the national healthcare system. The findings may help guide other centers considering the introduction of robotic thoracic surgery and contribute to the growing body of evidence on minimally invasive lung cancer treatment.

## 1. Introduction

Lung cancer accounts for the highest mortality rate among all cancers, responsible for approximately 18% of global cancer-related deaths [1]. The most common type is non-small cell lung cancer (NSCLC), accounting for approximately 85% of all lung cancer cases. For patients diagnosed with early-stage NSCLC, the standard therapeutic approach involves surgical excision of the affected lung tissue combined with the systematic removal of hilar and mediastinal lymph nodes. Among surgical techniques, lobectomy is widely regarded as the optimal procedure due to its balance of oncologic efficacy and preservation of pulmonary function [2]. Over the past two decades, minimally invasive techniques such as video-assisted thoracic surgery (VATS) have gained widespread acceptance in thoracic surgery due to their advantages in reducing operative trauma and shortening postoperative recovery times [3,4]. Robot-assisted thoracic surgery (RATS) represents a further evolution of minimally invasive surgical techniques. RATS enables surgeons to perform intricate dissections within anatomically challenging regions of the thorax, offering superior instrument maneuverability and ergonomic benefits compared to conventional approaches. Current research shows that RATS can have an advantage in number of dissected lymph nodes, shorter hospitalization duration and lower rate of postoperative complications [5,6,7,8]. Despite the growing body of evidence supporting RATS, several important limitations of the existing literature must be acknowledged. The majority of available data originate from high-volume centers with extensive minimally invasive experience, and it remains unclear whether these outcomes are reproducible during the early adoption phase at newly established programs [5]. Furthermore, most published series originate from North America or Western Europe, and data from Central and Eastern European healthcare settings—where access to robotic platforms has only recently expanded—remain virtually absent. Comparative studies suggest that RATS may offer advantages over VATS in terms of lymph node yield and conversion rate [6,7], yet direct evidence of its safety profile during program implementation, in unselected consecutive patients, is limited. In Poland, robotic thoracic surgery was introduced into routine clinical practice only within the last few years, and no published analyses exist describing perioperative outcomes or oncologic quality during this critical implementation period. This study addresses that gap by presenting the first single-surgeon experience with robotic-assisted lobectomy for NSCLC in the Polish healthcare system, with the aim of establishing initial benchmarks for perioperative safety, complication profiles, and oncologic adequacy that may guide future program development at other national centers.

## 2. Materials and Methods

This retrospective analysis of a prospectively maintained database included 89 consecutive patients who underwent robotic-assisted pulmonary lobectomy for suspected or histologically confirmed non-small cell lung cancer (NSCLC) at the Department of Thoracic Surgery, Military Medical Institute in Warsaw, between January 2022 and December 2024. The study period coincided with the institutional adoption of robotic thoracic surgery. All procedures were performed by a single thoracic surgeon using the da Vinci Xi Surgical System (Intuitive Surgical Inc., Sunnyvale, CA, USA).

All procedures were conducted using a standardized four-arm portal approach with an additional assistant port and carbon dioxide insufflation. The operative protocol included anatomical pulmonary lobectomy and systematic mediastinal lymphadenectomy, performed in accordance with institutional standards and international oncologic guidelines, including the European Society of Thoracic Surgeons (ESTS) and the International Association for the Study of Lung Cancer (IASLC) recommendations [9,10].

**Figure 1:**
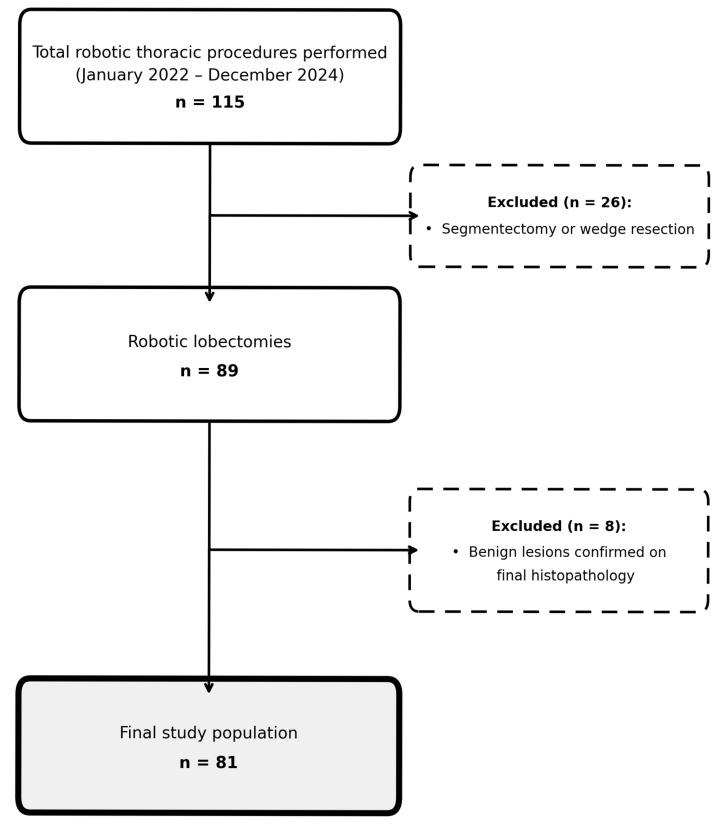
Patient selection flowchart.

A total of 115 robotic thoracic procedures were performed during the study period. Of these, 26 patients were excluded due to undergoing segmentectomy or wedge resection. Among the remaining 89 patients who underwent robotic lobectomy, 8 were further excluded because postoperative histopathological examination confirmed benign lesions. Consequently, 81 patients were included in the final analysis (see Figure 1).

Clinical and perioperative data were collected prospectively by the team and recorded in a structured Microsoft Excel database (Microsoft Corp., Redmond, WA, USA). Baseline characteristics included age, sex, body mass index (BMI), smoking history (current, former, never), and relevant comorbidities (hypertension, chronic obstructive pulmonary disease [COPD], heart failure, diabetes mellitus, hypothyroidism, renal failure, and cerebrovascular disease).

Perioperative outcomes included operative time (measured from skin incision to wound closure), intraoperative complications, conversion to thoracotomy, length of hospital stay, duration of chest tube drainage, and 30-day mortality. Postoperative complications were categorized according to the Clavien-Dindo classification system [11]. Each event was assigned a grade reflecting the most severe intervention required for its management. Postoperative events were recorded according to the standardized definitions provided by The Society of Thoracic Surgeons (STS) and the European Society of Thoracic Surgeons (ESTS) General Thoracic Surgery Databases [9]. Prolonged air leak (PAL), defined as air leak persisting beyond 5 days postoperatively, was managed conservatively with continued chest tube drainage in all cases [12].

Tumor characteristics were assessed based on final pathology reports. Parameters included tumor histologic subtype, pathologic staging according to the 8th edition of the IASLC TNM classification, resection margin status (R0 or R1), and the number of lymph nodes harvested. The mean total drainage volume refers to cumulative pleural drainage collected from the time of surgery until chest tube removal.

Formal approval from an ethics committee was not obtained due to the retrospective nature of the study. However, all patients signed informed consent upon admission to the hospital, authorizing the use of anonymized data for clinical and research purposes. The study was anonymous and performed in accordance with the ethical standards laid down in the 1964 Declaration of Helsinki and its latter amendments (Fortaleza).

Data analysis was performed using IBM SPSS Statistics for Windows, Version 29.0.2.0 (20) (IBM Corp., Armonk, NY, USA). Continuous variables were presented as means with standard deviations (SD) or medians with interquartile ranges (IQR), depending on distribution. Normality of continuous variables was assessed using the Shapiro–Wilk test. Categorical variables were reported as counts and percentages. No comparative statistical tests were performed due to the descriptive design of the study.

## 3. Results

A total of 81 patients underwent robotic-assisted lobectomy for NSCLC. The median age was 70 years (IQR: 65–74), with a slight male predominance (52%). Most patients had a history of smoking (67%). The most common comorbidities are detailed in Table 1.

The most frequently resected lobe was the right upper lobe (38%), followed by the left upper (25%), right lower (17%), left lower (15%), and right middle lobe (5%). The median operative time was 176 min (IQR: 149–220). The 90th percentile of operative time was 264 min. There were no intraoperative conversions to thoracotomy. One intraoperative complication occurred, involving a parenchymal injury to the left lung during thoracoscopic dissection, resulting in approximately 900 mL of blood loss. The bleeding was controlled intraoperatively without the need for conversion or transfusion, and it did not affect the postoperative course.

Postoperative complications occurred in 20 patients (24%) with the majority classified as Clavien-Dindo grade 1 or 2. The most common was prolonged air leak (>5 days) in 14 patients (17%), while 4 (5%) developed postoperative atrial fibrillation. Two patients (3%) required unexpected ICU admission due to postoperative respiratory failure: one with acute postoperative hypoxia, and another with pneumonia and atrial fibrillation, requiring intubation and mechanical ventilation. This second patient was discharged from the ICU after 23 days, with a total hospital stay of 43 days. Two patients (3%) were readmitted within 30 days. One was readmitted due to a bronchopleural fistula at the left upper lobe bronchial stump, while the other was hospitalized for exacerbation of a pre-existing chronic condition unrelated to the index surgery.

There were no 30-day mortalities. The median length of hospital stay was 8 days (IQR: 6–10). The median chest tube duration was 5 days (IQR: 3–7), and only one patient was discharged with a chest tube in place. The median total drainage volume was 440 mL (IQR: 180–735), representing cumulative pleural drainage from surgery until chest tube removal.

Pathologic examination confirmed R0 resection in 78 cases (96%), with three R1 resections in patients with locally advanced tumors. The most common histologic subtype was adenocarcinoma (51%), followed by squamous cell carcinoma (35%). Tumor staging revealed that 74% of patients were node-negative (pN0), 19% pN1, and 7% pN2. Pathologic T stages and full AJCC staging details are summarized in Table 1 and Table 2.

## 4. Discussion

Robotic-assisted thoracic surgery (RATS) has been increasingly adopted as a minimally invasive option in the surgical management of NSCLC. It offers enhanced stability, three-dimensional visualization, and greater ergonomic precision compared to conventional video-assisted thoracoscopic surgery. These advantages are particularly valuable in complex anatomic resections and mediastinal lymphadenectomy, where access can be limited. Numerous studies have demonstrated that RATS is associated with comparable, if not superior, perioperative outcomes, including reduced blood loss, shorter hospital stays, and lower conversion rates, while preserving oncologic rigor in terms of R0 resection rates and lymph node retrieval [6,13,14].

Robotic-assisted lobectomy has been investigated worldwide, with numerous single-institution series and large database analyses confirming its perioperative safety and oncologic adequacy. More than 200 single-arm studies and over 230 retrospective comparisons have evaluated the outcomes of robotic-assisted lobectomy. Despite this substantial global evidence, no corresponding data have been published from Poland. As robotic thoracic surgery was introduced into the Polish healthcare system only recently, this absence represents a significant national evidence gap. The present study provides the first detailed single-surgeon experience from a Polish center [15], establishing initial benchmarks for perioperative safety, complication profiles, and oncologic adequacy within a developing robotic surgery program. These findings may serve as a reference for future institutional implementations and contribute to shaping national standards for robotic thoracic surgery.

Although robotic-assisted lobectomy has gained increasing acceptance in thoracic surgery, the current evidence remains inconclusive regarding its specific advantages in patients with elevated surgical risk. Most available studies either do not stratify by comorbidity burden or are limited by retrospective designs and selection bias. In our cohort, a considerable number of patients presented with comorbid conditions such as COPD (24%), diabetes (15%), and cardiovascular disease (20%). Despite this, we observed a low complication rate and no 30-day mortality, suggesting that robotic lobectomy can be performed safely in a comorbid population. However, these findings should be interpreted cautiously, as the design and sample size do not permit direct comparison with other surgical modalities or definitive conclusions regarding differential benefit in high-risk patients.

No 30-day mortality or conversion to thoracotomy occurred. The overall complication rate of 24% aligns with rates reported in established robotic lobectomy series, supporting safety of the procedure during early adoption [13,16]. The most common complication was prolonged air leak, occurring in 17% of patients. Unexpected ICU admission occurred in only 3% of patients, and the readmission rate within 30 days was similarly low (3%). Chest tubes were still in place at discharge in one case, and 4% of patients experienced postoperative atrial fibrillation.

These findings support the procedural safety profile of RATS, especially given the complexity of oncologic resections and the presence of significant comorbidities in our cohort. Severe complications were infrequent, and the median hospital stay was 8 days (IQR: 6–10). These results suggest that robotic lobectomy can be performed with acceptable morbidity even during the early learning curve. These results also align with prior studies emphasizing RATS as a safe alternative to VATS, particularly in centers with growing robotic programs [6,17,18,19]. Our median operative time of 176 min is well within the range reported in recent robotic lobectomy series [17,20,21], which documented median durations between 132 and 199 min. Our overall complication rate of 24% was within the lower range to published benchmarks ranging from 20% to 35% across robotic series [22,23]. Despite being conducted during the early phase of learning curve, the results were equivalent to those reported in experienced centers. Although a direct comparison with VATS or open thoracotomy was beyond the scope of this descriptive study, the observed outcomes are broadly consistent with those reported for established minimally invasive techniques in the literature [6,17].

**Figure 2:**
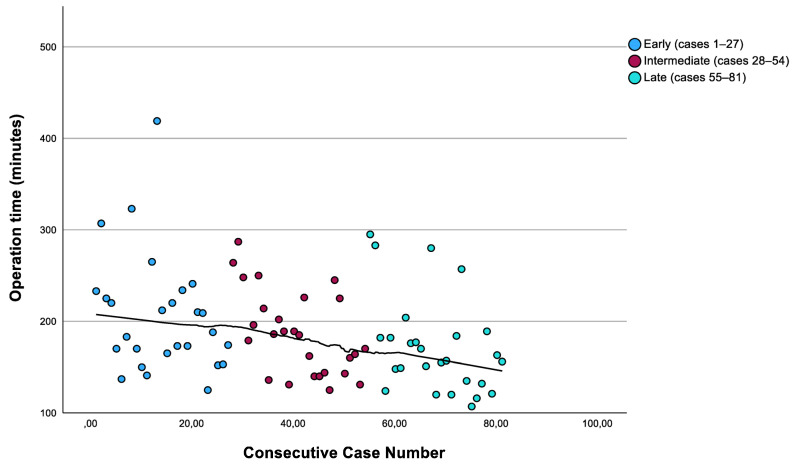
Operative time (minutes) across 81 consecutive robotic lobectomy cases. Each point represents an individual case, with marker color indicating the chronological cohort (Early: cases 1–27; Intermediate: cases 28–54; Late: cases 55–81). The black curve represents a LOESS trend line, illustrating the progressive reduction in operative time over the study period.

To provide an exploratory assessment of the learning curve, operative times were analyzed across three consecutive chronological cohorts of 27 patients each. The median operative time was 188 min (IQR: 165–233) in the early phase (cases 1–27), 185 min (IQR: 143–225) in the intermediate phase (cases 28–54), and 157 min (IQR: 132–184) in the late phase (cases 55–81). The temporal trend in operative time across consecutive cases is illustrated in Figure 2. This progressive reduction in operative time suggests a measurable learning effect over the study period, consistent with patterns described in other single-surgeon robotic lobectomy series during early program implementation [24,25]. While no formal learning curve analysis (e.g., cumulative sum (CUSUM) methodology) was performed, the observed temporal trend supports the feasibility of skill acquisition within the robotic thoracic surgery program. A formal analysis was not performed because the primary aim of this study was to describe perioperative and oncologic outcomes rather than to formally model the learning curve. Additionally, the sample size of 81 cases may be insufficient for a reliable CUSUM analysis, which typically requires a larger number of procedures and predefined failure thresholds.

Oncologic quality is a central measure in evaluating the effectiveness of lobectomy for non-small cell lung cancer, with particular emphasis on the extent of lymphadenectomy and status of resection margins. In our series, the median number of lymph nodes harvested was 14, from 5 distinct nodal stations. This meets, and in many cases exceeds, the lymphadenectomy standards outlined by international bodies. For example, current guidelines define complete resection as requiring systematic nodal dissection including assessment of at least six nodal stations, comprising at least three mediastinal stations with mandatory inclusion of the subcarinal node (station 7) [10,26]. Additional recommendations suggest that removal of ≥9 lymph nodes from at least 4 stations should be standard for stage IA2 and IA3 disease [27]. In our cohort, this threshold was achieved in 66 patients (81%), suggesting that robotic-assisted lobectomy may facilitate adherence to oncologic standards in early-stage NSCLC. Systematic lymphadenectomy was a standard component of every procedure, reflecting the principle that anatomical resection must be combined with comprehensive nodal assessment for precise staging and potential survival benefit [28].

Notably, 96% of patients in our cohort achieved an R0 resection, indicating clear surgical margins. This rate is on par with those reported in large robotic lobectomy series, where the R0 rate was between 94.4% and 98.4% [13,16]. Although long-term survival data were not captured in our series, existing literature suggests that both the extent of lymphadenectomy and margin status are positively associated with disease-free and overall survival in early-stage NSCLC [29,30]. The consistently negative margins observed even during the early learning curve further support the oncologic integrity of the robotic approach, comparable to open and VATS techniques [31].

This study offers a number of methodological strengths that enhance the interpretability of its findings. The prospective collection of complete perioperative, pathologic, and complication data allows for a granular analysis of clinical outcomes. All procedures were performed consecutively without exclusion of patients based on comorbidity burden or tumor location, which enhances the generalizability of the results within the context of single-surgeon experience. The study also demonstrates high adherence to international oncologic standards for lymph node dissection and resection margins, supporting the oncologic validity of the robotic approach during the early learning phase. Another strength of this study is that it addresses the current evidence gap within the Polish healthcare system.

Nevertheless, certain limitations must be acknowledged. As a descriptive study without multivariable analysis or propensity score adjustment, confounding variables were not formally controlled. This reflects the analytical approach rather than the study design per se, as retrospective datasets can accommodate confounder-adjustment methods. No direct comparison was made with alternative surgical modalities such as video-assisted thoracoscopic or open lobectomy. As a single-institution experience, the findings may not be fully generalizable to centers with differing surgical volume, patient demographics, or institutional protocols. Furthermore, it should be acknowledged that patients in this series were individually qualified for robotic surgery based on performance status and anatomical considerations, introducing a degree of selection bias inherent to surgical case series. Although a temporal analysis of operative times suggests a progressive reduction across three consecutive cohorts, a formal learning curve analysis using CUSUM or moving average methodology was not performed. Complication rates and oncologic outcomes were not assessed across time periods, and future dedicated studies should incorporate validated learning curve methodologies [24]. The absence of long-term oncologic outcomes, including disease-free survival and overall survival, represents a significant limitation that precludes conclusions regarding oncologic efficacy beyond the short-term period.

## 5. Conclusions

Our single-surgeon, single-institution experience demonstrates that robotic-assisted lobectomy for NSCLC is a feasible surgical approach with acceptable perioperative morbidity, even during the early phase of program implementation. From the perspective of the Polish healthcare system, these findings provide the first real-world evidence supporting the feasibility of introducing robotic thoracic surgery into routine clinical practice. The procedure was associated with low complication rates, no intraoperative conversions, and high rates of complete (R0) resection. Systematic mediastinal lymphadenectomy was feasible and consistent, meeting international oncologic standards in the majority of cases. Given the descriptive, single-center design, these findings should be interpreted with caution and require validation in larger, multicenter prospective studies.

These results highlight the procedural reliability and oncologic adequacy of the robotic approach in thoracic oncology. While long-term survival data are still needed, our findings support the integration of robotic lobectomy into routine clinical practice for appropriately selected NSCLC patients in Poland.

## Figures and Tables

**Table 1:** Baseline patient characteristics (*n* = 81).

Variable	Value
Age (years)	
Median [IQR]	70 [65–74]
Sex, n (%)	
Male	42 (52)
Female	39 (48)
BMI (kg/m^2^)	
Mean (SD)	26.4 (4.1)
FEV1, % predicted	
Mean (SD)	85.7 (19.2)
Smoking status, n (%)	
Current smoker	39 (48)
Former smoker	15 (19)
Never smoker	27 (33)
Comorbidities, n (%)	
Hypertension	57 (70)
COPD	19 (24)
Heart failure	16 (20)
Diabetes mellitus	12 (15)
Hypothyroidism	10 (12)
Renal failure	5 (6)
Cerebrovascular disease	4 (5)
Histologic subtype, n (%)	
Adenocarcinoma	41 (51)
Squamous cell carcinoma	28 (35)
Large cell carcinoma	6 (7)
Other	6 (7)
Resected lobe, n (%)	
Right upper lobe (RUL)	31 (38)
Left upper lobe (LUL)	20 (25)
Right lower lobe (RLL)	14 (17)
Left lower lobe (LLL)	12 (15)
Right middle lobe (RML)	4 (5)
pT stage, n (%)	
T1a	8 (10)
T1b	17 (21)
T1c	10 (12)
T2a	29 (36)
T2b	8 (10)
T3	4 (5)
T4	5 (6)
pN stage, n (%)	
N0	60 (74)
N1	15 (19)
N2	6 (7)
Pathologic stage (AJCC 8th ed.), n (%)	
IA1	6 (7)
IA2	13 (16)
IA3	8 (10)
IB	23 (28)
IIA	9 (11)
IIB	13 (16)
IIIA	6 (7)
IIIB	2 (3)
IVA	1 (1)

BMI = body mass index; COPD = chronic obstructive pulmonary disease; FEV1 = forced expiratory volume in 1 s; IQR = interquartile range; SD = standard deviation; AJCC = American Joint Committee on Cancer.

**Table 2:** Perioperative data and postoperative outcomes (n = 81).

Variable	Value
Operation time (min)	
Median [IQR]	176 [149–220]
Lymph node stations dissected	
Median [IQR]	5 [4,5]
Lymph nodes dissected (total)	
Median [IQR]	14 [11–20]
Length of hospital stay (days)	
Median [IQR]	8 [6–10]
Chest tube duration (days)	
Median [IQR]	5 [3–7]
Total drainage volume (mL) ^a^	
Median [IQR]	440 [180–735]
Surgical margin, n (%)	
R0	78 (96)
R1	3 (4)
Conversion to thoracotomy, n (%)	0 (0)
Intraoperative complications, n (%)	1 (1)
Postoperative complications, n (%)	20 (24)
Clavien-Dindo Grade 1	11 (14)
Clavien-Dindo Grade 2	4 (5)
Clavien-Dindo Grade 3	4 (5)
Clavien-Dindo Grade 4	1 (1)
Clavien-Dindo Grade 5	0 (0)
Prolonged air leak (>5 days), n (%)	14 (17)
Postoperative atrial fibrillation, n (%)	4 (5)
Unexpected ICU admission, n (%)	2 (3)
30-day readmission, n (%)	2 (3)
**30-day mortality, n (%)**	0 (0)

^a^ Data available for 63 of 81 patients. ICU = intensive care unit; IQR = interquartile range.

## Data Availability

The datasets used and/or analyzed during the current study are available from the corresponding author on reasonable request.

## Abbreviations

The following abbreviations are used in this manuscript:

AJCC	American Joint Committee on Cancer
BMI	Body Mass Index
CA	California
CD	Clavien–Dindo (classification)
COPD	Chronic Obstructive Pulmonary Disease
CO_2_	Carbon Dioxide
ESTS	European Society of Thoracic Surgeons
PAL	Prolonged Air Leak
IASLC	International Association for the Study of Lung Cancer
ICU	Intensive Care Unit
IQR	Interquartile Range
NSCLC	Non-Small Cell Lung Cancer
RATS	Robot-Assisted Thoracic Surgery
SD	Standard Deviation
SPSS	Statistical Package for the Social Sciences
STS	Society of Thoracic Surgeons
TNM	Tumor–Node–Metastasis (classification system)
USA	United States of America
VATS	Video-Assisted Thoracoscopic Surgery
